# A machine learning-based risk stratification tool for in-hospital mortality of intensive care unit patients with heart failure

**DOI:** 10.1186/s12967-022-03340-8

**Published:** 2022-03-18

**Authors:** Cida Luo, Yi Zhu, Zhou Zhu, Ranxi Li, Guoqin Chen, Zhang Wang

**Affiliations:** 1 South China Normal University-Panyu Central Hospital Joint Laboratory of Basic and Translational Medical Research, Guangzhou Panyu Central Hospital, Guangzhou, 511400 Guangdong China; 2 School of Life Sciences, South China Normal University, Guangzhou, 510631 Guangdong China; 3 Department of Cardiology, Guangzhou Panyu Central Hospital, Guangzhou, 511400 Guangdong China

**Keywords:** Machine learning models, Heart failure, Extreme gradient boosting, Medical information mart for intensive care, Risk stratification

## Abstract

**Background:**

Predicting hospital mortality risk is essential for the care of heart failure patients, especially for those in intensive care units.

**Methods:**

Using a novel machine learning algorithm, we constructed a risk stratification tool that correlated patients’ clinical features and in-hospital mortality. We used the extreme gradient boosting algorithm to generate a model predicting the mortality risk of heart failure patients in the intensive care unit in the derivation dataset of 5676 patients from the Medical Information Mart for Intensive Care III database. The logistic regression model and a common risk score for mortality were used for comparison. The eICU Collaborative Research Database dataset was used for external validation.

**Results:**

The performance of the machine learning model was superior to that of conventional risk predictive methods, with the area under curve 0.831 (95% CI 0.820–0.843) and acceptable calibration. In external validation, the model had an area under the curve of 0.809 (95% CI 0.805–0.814). Risk stratification through the model was specific when the hospital mortality was very low, low, moderate, high, and very high (2.0%, 10.2%, 11.5%, 21.2% and 56.2%, respectively). The decision curve analysis verified that the machine learning model is the best clinically valuable in predicting mortality risk.

**Conclusion:**

Using readily available clinical data in the intensive care unit, we built a machine learning-based mortality risk tool with prediction accuracy superior to that of linear regression model and common risk scores. The risk tool may support clinicians in assessing individual patients and making individualized treatment.

**Supplementary Information:**

The online version contains supplementary material available at 10.1186/s12967-022-03340-8.

## Background

Heart failure is a complex clinical syndrome caused by structural or functional impairment of the heart [1, 2]. Heart failure has a high incidence in critically ill patients, especially among those in intensive care units (ICUs), and it is responsible for poor outcomes by causing myocardial injury and increased in-hospital mortality [3]. Critical-illness scoring systems, such as the acute physiology and chronic health evaluation-II (APHACHE-II) and the simplified acute physiology score-II (SAPS-II), have been widely used in critical care medicine. However, they have been only modestly successful in heart failure populations [4–6]. Nowadays, the prognosis for critical patients with advanced heart failure remains poor, and a proportion of patients require higher acuity care in the ICU. We need a more precise risk stratification tool to improve the quality of heart failure care in the ICU [7, 8]. On the other hand, traditional prediction models based on logistic regression analysis for heart failure, such as Get With Guidelines Heart Failure (GWTGW)-HF Registry, may not capture multi-dimensional correlations that contain prognostic information from large amounts of high dimensional data while we can get much characteristic information from the detection instrument in the ICU [9]. In contrast, novel machine learning techniques can capture the nonlinear relationship between patients’ prognosis and clinical manifestations and identify patterns from large datasets that have many variables [10–12]. Extreme gradient boosting (XGBoost) is an ensemble learning algorithm combining multiple machine learning algorithms serially to obtain a better model that can learn more complex decision boundaries and efficiently handle missing data [13]. XGBoost gained significant favor in the last few years due to helping individuals and teams win virtually every Kaggle structured data competition. What is more, XGBoost has had good performance in prognostic prediction models [14–16].

In this study, we used XGBoost methods to generate a more precise risk predictive model on in-hospital mortality among critically ill patients with heart failure compared with traditional prediction models and critical illness scoring systems. We further validated the machine learning model by plotting the decision curve and assessing predictive performance in external populations.

## Materials and methods

### Database

Two distinct databases were used for this study. The model was developed from a retrospective analysis of a cohort of patients from Medical Information Mart for Intensive Care (MIMIC-III) a large public database that includes information on 46,520 patients who were admitted to ICUs from 2001 to 2021 at the Beth Israel Deaconess Medical Center in Boston, MA, USA [17]. The database contains records of demographics, hourly vital signs from bedside monitors, laboratory tests, International Classification of Diseases and Ninth Revision (ICD-9) codes diagnoses, and other clinical characteristics. The users were required to pass a test to qualify to register for the database and to be approved by the MIMIC-III database administration staff. The second cohort of patients was from the Telehealth Intensive Care Unit (eICU) Collaborative Research Database (eICU-CRD) as a validation dataset. The eICU-CRD, a multi-center critical care database, covers more than 200,000 ICU stays of 139,367 unique patients admitted to ICUs between 2014 and 2015 from 208 hospitals in the United States [18]. After passing a training course, “Protecting Human Research Participants,” on the website of the National Institutes of Health, we had permission to extract data from the two databases for research purposes (certification number: 37903239).

### Study population

The study focused on ICU patients with heart failure. We exported the patients who were diagnosed with heart failure at admission to an ICU from the MIMIC-III and the eICU-CRD through ICD-9 codes or who were recorded as heart failure patients. Other criteria for inclusion were (I) heart failure without sepsis at admission to the ICU; (II) older than 16 years old and younger than 90 years old; (III) first hospital stay and the first ICU admission; IV) longer than 24-h stay in the ICU; (V) ICU vital signs data and laboratory test data available.

### Data extraction

Initially, we extracted as many features as possible for constructing the baseline model and feature screening from the MIMIC-III database. First, we collected demographic data, including age, gender, weight, height, and ethnicity. Then, the vital signs data and laboratory data during the first 24 h after admission to the ICU were extracted, including heart rate, blood pressure, respiratory rate, temperature, oxyhemoglobin saturation (SpO2), creatinine, chloride, glucose, hematocrit, hemoglobin, platelet count, potassium, partial thromboplastin time (PTT), prothrombin time (PT), sodium, blood urea nitrogen (BUN), white blood cell (WBC) count, red blood cell count, red cell distribution width (RDW), Pappenheimer O2 (pO2), partial pressure of carbon dioxide (pCO_2_), and HCO_3_. The clinicians and nurses collected these data hourly. For mining more information about these features, we took the maximum, minimum, mean, and range values of vital signs and laboratory data over a period as candidate features. Comorbidities of patients were also collected. The urine output and Glasgow Coma Scale were calculated in the first 24 h after ICU admission. The primary endpoint was all-cause in-hospital mortality, so patients without discharge information were excluded from the final cohort. Finally, these features were integrated into a single data frame for analysis. The data extraction process was conducted by use of the PostgreSQL programming language.

### Data preprocessing

After data extraction, the data set was preprocessed. The records with physiologically impossible values were eliminated. We then transformed character variables into categorical variables. If categorical variables were unordered, we coded them by One-Hot Encoding. Missing data, which were common in the databases, would introduce bias to subsequent analysis [19, 20]; to avoid introducing this bias, we excluded covariates with > 40% missing data and patients with > 20% missing covariates. In the missing data imputation stage, we compared three methods: (1) median imputation, (2) random forest imputation, and (3) Extreme gradient boosting (XGBoost) imputation. Since the XGBoost method had the best effect to predict in the baseline model, we selected it to handle the missing data.

### Model development

Generating the risk prediction model consisted of two stages: feature selection and model building. The feature selection stage selected the smallest and most predictive subset of features that were included in the final prediction model to minimize overfitting, as overfitting can lead to over-training of the training cohort and loss of prediction power in other populations. We used the permutation-based XGBOOST selection method, which ranks features by the variable importance metric of the XGBOOST and eliminated features one by one to get the best predictive subset (details in Additional file 1: Fig. S2).

Since the aim was to provide decision-making support for clinicians in evaluating the risk of in-hospital mortality of heart failure patients after ICU admission, the primary outcome of the model was the mortality rate of the ICU patients. The machine learning model was developed with the XGBoost algorithm [21, 22]. The algorithm was dependent on continuous iterative correction of residuals from previous weak models, meaning that the current classifier is determined based on the previous classifier to optimize predictive power [23, 24]. The MIMIC-III dataset provides more detailed information than the eICU dataset: First, through data preprocessing, the number of candidate feature set in the MIMIC-III dataset is 177, while the eICU is 89. All the features in eICU were incorporated in the MIMIC-III dataset, whereas the MIMIC-III dataset contains additional features regarding blood gas analysis and comorbidity information, such as arterial base excess, plasma bicarbonate, hematocrit, chronic pulmonary heart disease, valvular disease, pulmonary circulation, hypothyroidism and so on. Second, the size of the study cohort of the MIMIC-III dataset is 5676, while the eICU is 1349. In order to construct superior models and explore the most discriminating subset of variables, we used the MIMIC-III dataset as derivation data. We randomly divided the derivation data into a training cohort (90%) and a testing cohort (10%). The training cohort was used to train the predictive model, and the testing cohort was used to validate the performance of the predictive model. To train the machine learning model, we used the tenfold cross validation method in the training cohort for model hyperparameter tuning [25]. We used the best predictive model and calculated the area under the receiver operating characteristic curves (AUC) in the testing cohort. We also constructed other models (logistic regression and SAPS-II) to compare with the machine learning model in the testing cohort. For logistical regression, we constructed a new feature set by variable interactions. Then, the performance of stepwise logistical regression, Lasso, Ridge and Elastic Net was compared between the original feature set and the new feature set (details in Additional file 1: Fig. S2). The stepwise logistic regression model was conducted using these significant variables identified by forward stepwise analysis with each variable iteratively added to minimize the Akaike Information Criterion (AIC). Finally, the best model was selected and compared with the machine learning model. The data extraction process and model building were conducted with Python 3.8.3.

## Results

### Statistical analysis

A total of 5676 patients diagnosed with heart failure by MIMIC-III met our selection criteria. The selection cohort was divided into two groups based on whether they survived before discharge. Their data were presented by continuous variables (as means and standard deviation) or categorical variables (as frequencies and percentages) (Table 1). To identify the differences, the Kolmogorov–Smirnov test was used for continuous variables of normal distribution, and the Mann–Whitney U test was used for continuous variables of non-normal distribution. The differences of categorical variables between groups were tested with a Chi-squared test. The mean length of stay in the ICU was 5.1 days, and 595 patients died in the ICU, which was 10.5% of the deviation dataset. The patients who died in the hospital were older and had a lower BMI (p < 0.01) than did those who survived (Table 1). Other differences between the patients who survived and those who died are also given in Table 1).

**Table 1:** Baseline characteristics, vital signs, and laboratory test results of survivors compared with patients who died

Variables	Survived (n = 5081)	Died (n = 595)	p
Age, mean (years, SD)	70.2 (12.7)	74.5 (11.8)	< 0.001
Gender, n (%)	2842 (55.9%)	319 (53.6%)	0.281
BMI, mean (kg/m^2^, SD)	29.3 (7.5)	28.4 (7.9)	< 0.001
Heart rate, mean (bpm, SD)	84.0 (16.2)	86.9 (17.3)	< 0.001
SBP, mean (mmHg, SD)	117.6 (16.2)	115.7 (19.1)	0.002
DBP, mean (mmHg, SD)	58.5 (10.0)	56.8 (10.9)	< 0.001
Mean bp, mean (mmHg, SD)	76.7 (10.1)	75.6 (9.8)	0.014
Respiratory rate, mean (/min, SD)	19.0 (3.8)	20.3 (4.7)	< 0.001
Temperature, mean (°C, SD)	36.8 (0.6)	36.7 (0.8)	0.011
SpO2, mean (%, SD)	97.1 (2.0)	96.7 (2.9)	0.039
GCS, mean (SD)	12.1 (3.3)	10.3 (4.0)	< 0.001
Anion gap, mean (mmHg, SD)	13.8 (3.2)	15.9 (3.9)	< 0.001
HCO3, mean (mmol/L, SD)	24.8 (4.6)	23.6 (5.4)	< 0.001
Creatinine, mean (μmol/L, SD)	1.5 (1.4)	1.9 (1.4)	< 0.001
Chloride, mean (mmg/dL, SD)	104.5 (5.7)	103.7 (6.1)	0.004
Glucose, mean (mg/dL, SD)	144.1 (48.8)	160.7 (64.2)	< 0.001
Hematocrit, mean (%, SD)	31.8 (5.3)	31.9 (5.3)	0.358
Hemoglobin, mean (g/dL, SD)	10.7 (1.8)	10.6 (1.8)	0.961
Platelets, mean (× 10^9^/L, SD)	213.6 (96.7)	208.2 (104.8)	0.174
PTT, mean (s, SD)	41.4 (21.3)	46.6 (26.1)	< 0.001
INR, mean (SD)	1.5 (0.8)	1.7 (1.0)	< 0.001
PT, mean (s, SD)	16.0 (6.1)	17.2 (7.4)	< 0.001
Sodium, mean (mmol/L, SD)	138.2 (4.0)	138.6 (4.9)	0.002
BUN, mean (mmol/L, SD)	29.8 (21.9)	42.0 (27.0)	< 0.001
WBC, mean (× 10^9^/L, SD)	12.0 (7.0)	14.3 (20.0)	< 0.001
MCHC, mean (× 10 g/L, SD)	33.8 (1.5)	33.3 (1.5)	< 0.001
RBC, mean (× 10^9^/L, SD)	3.6 (0.6)	3.6 (0.7)	0.246
RDW, mean (%, SD)	15.1 (1.9)	15.8 (2.2)	< 0.001
Ph blood, mean (SD)	7.4 (0.1)	7.4 (0.1)	0.239
PO_2_, mean (mmHg, SD)	156.0 (73.8)	137.9 (74.4)	< 0.001
PCO_2,_ mean (mmHg, SD)	42.8 (9.7)	42.0 (11.5)	< 0.001
Urine output, mean (mL, SD)	1963.2 (1160.0)	1442.1 (1126.5)	< 0.001
Comorbidities			
Cardiac arrhythmias, n (%)	2958 (58.2)	377 (63.4)	0.016
Hypertension, n (%)	3187 (62.7)	299 (50.3)	< 0.001

*SD* standard deviation, *BMI* body mass index, *SBP* systolic blood pressure, *DBP* diastolic blood pressure, *SpO2* Oxygen saturation, *GCS*, Glasgow Coma scale, *PTT* partial thromboplastin time, *INR* international normalized ratio, *PT* prothrombin time, *BUN* blood urea nitrogen, *WBC* white blood cell count, *MCHC* mean corpuscular hemoglobin concentration, *RBC* red blood cell count, *RDW* red blood cell distribution width, *Ph* potential hydrogen, *PO2* partial pressure of arterial oxygen, *PCO2* partial pressure of arterial carbon dioxide

### Features selected in models

**Fig. 1:**
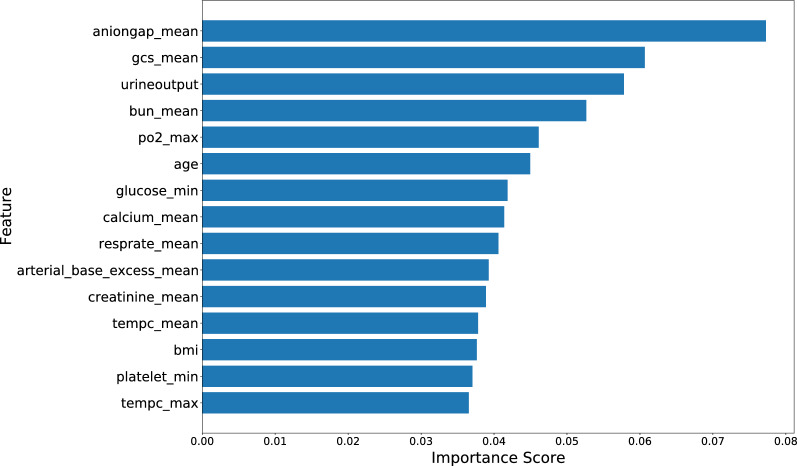
Feature importance derived from the XGBoost model

Through the feature screening stage, 24 features were selected in the final model. The cross validation AUC score declined slowly before the feature set was 24 (details in Additional file 1: Fig. S1). We used the XGBoost model to rank each features’ contribution for predicting. Mean anion gap, mean Glasgow Coma scale, urine output, mean BUN, maximum pO2, age, minimum glucose, mean calcium, mean respiratory rate, mean arterial base excess, mean creatinine, mean temperature, BMI, minimum platelet and maximum temperature were the top 15 most important features from the predictive models (Fig. 1).

### Internal validation and model comparisons

**Fig. 2:**
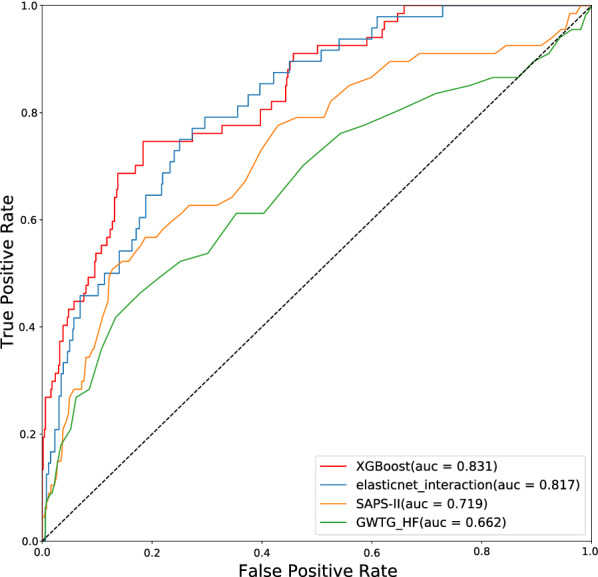
The receiver operating characteristic curves of the XGBoost model, elastic net model, SAPS-II score, and GWTG-HF score

**Fig. 3:**
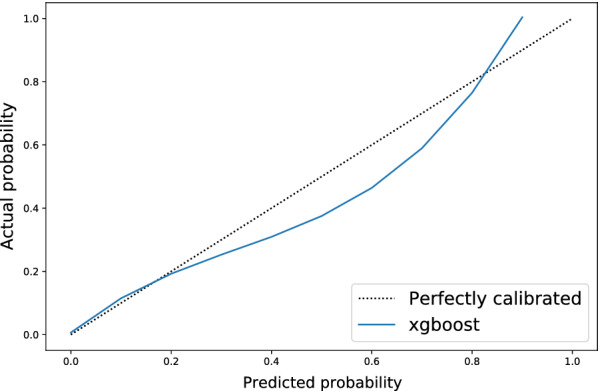
Calibration plot for the XGBoost model. The model had good calibration with in-hospital mortality risk

In internal validation, the GWTG-HF, SAPS-II, logistic regression, and XGBoost model had discriminator performance with AUC of 0.667 (95% CI 0.656–0.678), 0.72 (95% CI 0.710–0.736), 0.817 (95% CI 0.798–0.835) and 0.831 (95% CI 0.820–0.843), respectively (Fig. 2). The XGBoost model had better predictive power than did the others. The calibration plots of the XGboost model are described in Fig. 3, which agreed well with the validation cohort.

**Fig. 4:**
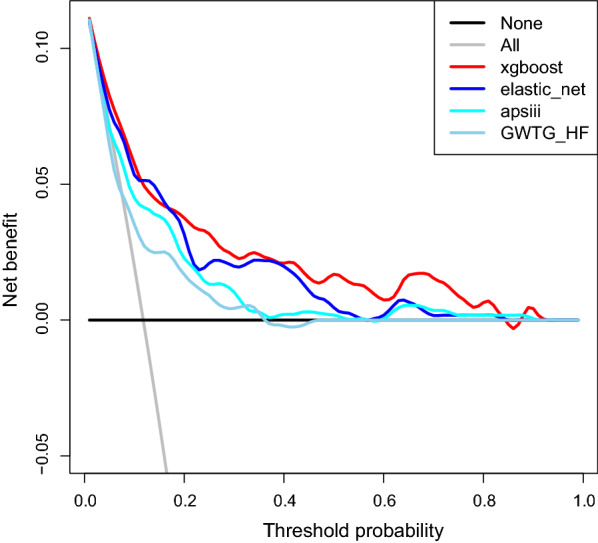
Decision curve analysis of models. The X axis indicates the threshold probability for in-hospital mortality, and the Y axis indicates the net benefit

Using the risk predictive model, we determined the risk probability stratification of heart failure patients in the testing dataset (Table 2). In that dataset, 60.3% of patients had a risk of 10% or less, which corresponded to a low hospital mortality rate. Moderate risk strata (10–30% predictive risk), high risk strata (30–50% predictive risk), and very high-risk strata (> 50% predictive risk) were present in 11.5%, 21.2%, and 56.2% hospital-mortality rate, respectively. The decision curve analysis of four models is illustrated in Fig. 4, in which the threshold risk probability of patients is about 10–80%. The XGBoost model to predict patients in-hospital mortality had more benefits than the treat-none strategy or the treat-all-patients strategy. The net benefit for the XGBoost model was more significant than other models, suggesting the XGBoost model was optimal.

**Table 2:** Rates of mortality in 5 different risk strata predicted by the XGBoost model in the internal validation dataset (n = 568)

Risk strata	Predictive hospital-mortality risk (%)	Rate of total study population (%)	Hospital-mortality (%)
Very low	≤ 5	245 (43.1%)	2.0
Low	5–10	98 (17.2%)	10.2
Moderate	10–30	130 (22.9%)	11.5
High	30–50	47 (8.3%)	21.2
Very high	> 50	48 (8.5%)	56.2

### External validation

We further validated the XGBoost model in the external dataset by using the eICU database with the same data extraction process as the derivation dataset. The main baseline variables of the two datasets are summarized in Table 3. Among 50 features selected by logistic regression, 18 (36%) features were not available in the eICU dataset. In comparison, for the XGBoost model, 24 features were selected and all but one features (arterial base excess) were available in the eICU dataset. Therefore, we consider it suboptimal to apply the logistic regression to the validation cohort. Since the arterial base excess feature was not available in the eICU database, we imputed the values of this feature by a regression model, which was constructed by the final feature set and the derivation dataset. We also performed an imputation by the regression analysis, which was constructed by the final feature set and the derivation dataset. We then evaluated the performance of the XGBoost model on this new dataset. The XGBoost model had a slight deterioration of performance, with an AUC of 0.826 (95% CI 0.805–0.847) in this dataset. The AUC in the external validation dataset was 0.809 (95% CI 0.805–0.814). Using the risk predictive model, we determined the risk probability stratification of heart failure patients in the external validation dataset (Table 4). The observed in-hospital mortality rates of very low, low, moderate, high, and very high risk strata were 3.2%, 5.6%, 19.5%, 41.0% and 53.7%, respectively. Thus, the XGBoost model also had good predictive performance in independent external populations. However, the robustness of the XGBoost model needs further clinical evaluation with other populations.

**Table 3:** Baseline patient characteristics between MIMIC-III and eICU

Variables	MIMIC-III (n = 5676)	eICU (n = 1349)
Age, mean (years, SD)	70.7 (12.6)	67.8 (13.7)
Men, n (%)	3161 (55.7)	802 (59.5)
BMI, mean (kg/m^2^, SD)	29.2 (7.5)	31.5 (12.9)
SBP, mean (mmHg, SD)	117.4 (16.5)	119.1 (19.8)
PTT, mean (s, SD)	41.9 (21.9)	42.0 (18.7)
Temperature, mean (°C, SD)	36.8 (0.6)	36.7 (0.6)
GCS, mean (SD)	11.9 (3.4)	13.4 (2.7)
Respiratory rate, mean (/min, SD)	19.1 (3.9)	20.3 (4.1)
Phosphate, mean (mg/dL, SD)	3.8 (1.3)	4.1 (1.4)
Calcium, mean (mmol/L, SD)	8.4 (0.8)	8.5 (0.7)
Glucose, mean (mg/dL, SD)	145.8 (50.9)	148.6 (58.3)
Urine output, mean (mL, SD)	1908.6 (1167.4)	1837.4 (1408.6)
Arterial base excess, mean (mmol/L, SD)	0.03 (4.3)	NA
Mortality (%)	10.5	12.8

*SD* standard deviation, *BMI* body mass index, *SBP* systolic blood pressure, *PTT* partial thromboplastin time, *GCS* Glasgow Coma Scale, *bp* blood pressure, *NA* not available

**Table 4:** Rates of mortality in 5 different risk strata predicted by the XGBoost model in the external validation dataset (n = 1060)

Risk Stata	Predictive hospital-mortality risk (%)	Rate of total study population (%)	Hospital-mortality (%)
Very low	≤ 5	532 (50.2%)	3.2
Low	5–10	195 (34.3%)	5.6
Moderate	10–30	231 (21.8%)	19.5
High	30–50	61 (10.7%)	41.0
Very high	> 50	41 (7.2%)	53.7

## Discussion

In this work, we used innovative machine-learning to construct a risk predictive model for hospital mortality among heart failure patients in intensive care units. Compared with traditional risk prediction, machine-learning techniques can capture the nonlinearity between risk predictors and mortality from large amounts of high dimensional data [26–28]. The techniques can overcome the challenge of accurately identifying high-risk patients in the ICU, especially for those with complex phenotypes, such as heart failure [29]. Matthew et al. [30] demonstrated the superiority of machine learning methods to predict the risk of heart failure. Our machine learning model had the best ability to distinguish among the three predictive models, with an AUC of 0.831 in the internal validation dataset. According to the DCA of the three models, the net benefit for the XGBoost model was maximum, suggesting that the XGBoost model is optimal. It also had acceptable performance, with an AUC of 0.809 (95% CI 0.805–0.814) in the external validation. The XGBoost model had satisfactory calibration and good risk stratifying ability both in the internal testing dataset and the external validation dataset.

Using the XGBoost model, we divided the risk probabilities into < 5%, 5–10%, 10–30%, 30–50%, > 50% as very low, low, moderate, high, and very high-risk strata in the derivation population, respectively. In addition, the risk strata were presented in the external validation dataset. We documented the feasibility of the XGBoost model to distinguish risk patients from other populations. Through the use of the XGBoost model, the risk probability of each patient can inform and support clinicians in decision making. However, there were some deaths in low-risk strata and some survivors in high-risk strata. We suspect that these exceptions may be due to different phenotypes of heart failure patients in various risk stratification. For instance, Matthew et al. [31] identified phenogroups of patients with machine learning-based unsupervised cluster analysis. Consequently, we may use other methods for further analysis and for making experimental validations in future research.

The machine learning-based model identified 24 variables from the feature set. Anion gap was most associated with death among ICU heart failure patients through the predictive model. Age was generally associated with death, and the Glasgow Coma Scale was also a predictor of mortality in ICU patients. Blood coagulation status at ICU admission, such as platelet count and PTT, was associated with in-hospital mortality among heart failure patients. Disturbance of blood coagulation has been reported to seriously threaten patients’ survival [32]. However, most heart failure patients receive anticoagulant therapy, which will add to coagulation abnormalities. Hence, clinicians should be cautious in prescribing anticoagulant therapy for patients who are at high risk because the agents may increase the risk of inducing coagulopathy. In order to implement faster and more accurate coagulation management, we could early implement thromboelastography (TEG) or rotational thromboelastometry (ROTEM) to high-risk patients [33]. Furthermore, the high-risk patients may receive mechanical thromboprophylaxis with intermittent pneumatic compression, graduated compression stockings, or percutaneous left atrial appendage closure [34, 35]. The volume of urine output was the third important predictor in the predictive model, and a higher volume of urine output may indicate a better prognosis. Lin et al. [36] indicated that decreased urine output could be a compensatory mechanism to maintain intravascular volume, and in that circumstance, patients may be at risk of renal injury. Meanwhile, oliguria and worsening renal function may drive fluid retention increasing the burden on the heart, which causes damage to the heart and aggravates symptoms of heart failure. Several studies in HF patients have demonstrated that fluid overload is independently associated with increased mortality [37, 38]. One reason was that HF patients are at risk of death not only from cardiovascular disease but also from multiorgan failure. Many features in blood gas analysis were among the most important features from the predictive models: pO2, pCO2, anion gap, and arterial base excess. However, through the machine learning method, we could only appreciate that heart failure was associated with these features; the method could not explain the mechanisms responsible for heart failure. Hence, further research is needed to determine the role of these features in ICU patients with heart failure.

As a retrospective analysis, this study has limitations. First, our predictive model was constructed from a single-center dataset, which may not be appropriate for other populations. Although our model had good performance in the external dataset, it needs verification in other datasets and populations. Second, because of missing data, some features that have been identified as risk predictors of heart failure, such as N-terminal pro-B-type natriuretic peptide [39, 40], were not assessed. Third, we did not make the most of time sequence data monitoring from the ICU; we only extracted the minimum, maximum, mean, and range of features within 24 h. The pattern of change for a period in a feature may contain information that can increase the prediction and understanding of mechanisms. In future work, we could divide the 24 h into shorter time intervals. One strategy is that the 24 h period can be divided into two time periods according to the maximum or minimum point of each time series feature. Then, we could extract additional summary statistics of the feature for the two time periods, such as mean value, variance, deviation and Shannon entropy, and incorporate them in the statistical models [41]. Nonetheless, our model can help clinicians identify heart failure in ICU patients who are at high risk for in-hospital mortality.

## Conclusions

This study showed that machine-learning algorithms can generate a high-performance risk-prediction tool for patients with heart failure in the ICU. The machine-learning algorithms monitor patients’ clinical data without requiring specific cardiovascular biomarkers and survival of different stages when integrated into electronic health record systems. The risk-prediction model can support clinicians in assessing heart failure patients in the ICU and in making personalized treatment plans. However, this application needs to be validated in the study of more independent cohorts.

## Supplementary Information

**Additional file 1: Figure S1.** The AUC of feature screening with fivefold CV. The vertical dotted line represents the number of features where the hyperparameter tuning was performed. **Figure S2.** Feature importance derived from XGBoost model when the feature set was 177. **Figure S3.** Feature importance derived from XGBoost model when the feature set was 86. **Figure S4.** Feature importance derived from XGBoost model when the feature set was 54. **Figure S5.** The receiver operating characteristic curves of the eight models.

## Data Availability

The datasets used and/or analyzed during the current study are available from the corresponding author on reasonable request.

## Abbreviations

ICU: Intensive care unit
HF: Heart failure
APHACHE-II: Acute physiology and chronic health evaluation-II
SAPS-II: Simplified acute physiology score-II
GWTGW-HF: Get With Guidelines Heart Failure
XGBoost: Extreme gradient boosting
LR: Logistic regression
MIMIC-III: Medical Information Mart for Intensive Care III
ICD-9: International Classification of Diseases and Ninth Revision
eICU-CRD: Telehealth Intensive Care Unit Collaborative Research Database
SD: Standard deviation
BMI: Body mass index
SBP: Systolic blood pressure
DBP: Diastolic blood pressure
SpO2: Oxygen saturation
GCS: Glasgow Coma scale
PTT: Partial thromboplastin time
INR: International normalized ratio
PT: Prothrombin time
BUN: Blood urea nitrogen
WBC: White blood cell count
MCHC: Mean corpuscular hemoglobin concentration
RBC: Red blood cell count
RDW: Red blood cell distribution width
Ph: Potential hydrogen
PO2: Partial pressure of arterial oxygen
PCO2: Partial pressure of arterial carbon dioxide
Max: Maximum
Min: Minimum
AUC: Area under curves
ROC: The receiver operating characteristic curves
NA: Not available
