# Supplementary material for: A machine learning-based risk stratification tool for in-hospital mortality of intensive care unit patients with heart failure
Source: J Transl Med. 2022 Mar 18;20:136. doi: 10.1186/s12967-022-03340-8 (PMC8932070; doi:10.1186/s12967-022-03340-8)
Supplement: Supplementary file 1 — Additional file 1: Figure S1. The AUC of feature screening with fivefold CV. The vertical dotted line represents the number of features where the hyperparameter tuning was performed. Figure S2. Feature importance derived from XGBoost model when the feature set was 177. Figure S3. Feature importance derived from XGBoost model when the feature set was 86. Figure S4. Feature importance derived from XGBoost model when the feature set was 54. Figure S5. The receiver operating characteristic curves of the eight models. [file 12967_2022_3340_MOESM1_ESM.docx]

**A Machine Learning-based Risk Stratification Tool for In-hospital Mortality of Intensive Care Unit Patients with Heart Failure**

**Additional Material**

**1.Feature Selection**

We used the permutation-based XGBOOST selection method, which ranks features by the variable importance metric of the XGBOOST and eliminated features one by one to get the best predictive subset. We performed hyperparameter tuning along with feature selection on a regular basis. Once the decline in accuracy exceeded our predetermined threshold, which is that the score differs more than 1% from the base model, we stopped feature selection and performed hyperparameter tuning. After doing hyperparameter tuning, we recalculated the importance of features and proceeded with feature selection. The process was reiterated until no more variables are eligible for elimination in the model. Based on this rule, the hyperparameter tuning was performed when the feature numbers were 86, 54, and 24 respectively. The final feature set was 24. The 24 features were included: Mean anion gap, mean Glasgow Coma scale, urine output, mean BUN, maximum pO2, age, mean calcium, minimum glucose, mean respiratory rate, mean arterial base excess, mean creatinine, BMI, mean temperature, mean magnesium, maximum temperature, maximum platelet, minimum PT, mean SBP, mean PTT, mean spO2, mean PT, mean DBP and minimum PTT. The AUC changing with feature numbers is shown in Figure 1. The top 15 most important features in feature set 177, 86 and 54 were shown in Figure 2, 3 and 4.

Figure S1. The AUC of feature screening with fivefold CV. The vertical dotted line represents the number of features where the hyperparameter tuning was performed.

Figure S2. Feature importance derived from XGBoost model when the feature set was 177.

Figure S3. Feature importance derived from XGBoost model when the feature set was 86.

Figure S4. Feature importance derived from XGBoost model when the feature set was 54.

**2.Logistical Regression**

With regard to logistical regression, we constructed a new feature set by variable interactions-we combinate variables by multiplying and taking the ratio between different features. The stepwise logistical regression, Lasso, Ridge and Elastic Net on the original feature set and the new feature set was test in the testing cohort with ROC curves in Figure 5.

Figure S5. The receiver operating characteristic curves of the eight models.
